# Infrared Spectral Signatures of Nucleobases in Interstellar Ices I: Purines

**DOI:** 10.3390/life13112208

**Published:** 2023-11-14

**Authors:** Caroline Antunes Rosa, Alexandre Bergantini, Péter Herczku, Duncan V. Mifsud, Gergő Lakatos, Sándor T. S. Kovács, Béla Sulik, Zoltán Juhász, Sergio Ioppolo, Heidy M. Quitián-Lara, Nigel J. Mason, Claudia Lage

**Affiliations:** 1 Carlos Chagas Filho Institute of Biophysics, Federal University of Rio de Janeiro, Rio de Janeiro 21941-170, Brazil; 2 Celso Suckow da Fonseca Federal Centre for Technological Education, Rio de Janeiro 20271-110, Brazil; 3 HUN-REN Institute for Nuclear Research (Atomki), H-4026 Debrecen, Hungary; 4 Institute of Chemistry, University of Debrecen, H-4032 Debrecen, Hungary; 5 Centre for Interstellar Catalysis (InterCat), Department of Physics and Astronomy, University of Aarhus, DK-8000 Aarhus, Denmark; 6 Centre for Astrophysics and Planetary Science, School of Physics and Astronomy, University of Kent, Canterbury CT2 7NH, UK

**Keywords:** astrobiology, astrochemistry, purines, nucleobases, adenine, guanine, interstellar medium, infrared spectroscopy

## Abstract

The purine nucleobases adenine and guanine are complex organic molecules that are essential for life. Despite their ubiquitous presence on Earth, purines have yet to be detected in observations of astronomical environments. This work therefore proposes to study the infrared spectra of purines linked to terrestrial biochemical processes under conditions analogous to those found in the interstellar medium. The infrared spectra of adenine and guanine, both in neat form and embedded within an ice made of H_2_O:NH_3_:CH_4_:CO:CH_3_OH (10:1:1:1:1), were analysed with the aim of determining which bands attributable to adenine and/or guanine can be observed in the infrared spectrum of an astrophysical ice analogue rich in other volatile species known to be abundant in dense molecular clouds. The spectrum of adenine and guanine mixed together was also analysed. This study has identified three purine nucleobase infrared absorption bands that do not overlap with bands attributable to the volatiles that are ubiquitous in the dense interstellar medium. Therefore, these three bands, which are located at 1255, 940, and 878 cm^−1^, are proposed as an infrared spectral signature for adenine, guanine, or a mixture of these molecules in astrophysical ices. All three bands have integrated molar absorptivity values (*ψ*) greater than 4 km mol^−1^, meaning that they should be readily observable in astronomical targets. Therefore, if these three bands were to be observed together in the same target, then it is possible to propose the presence of a purine molecule (i.e., adenine or guanine) there.

## 1. Introduction

**Figure 1:**
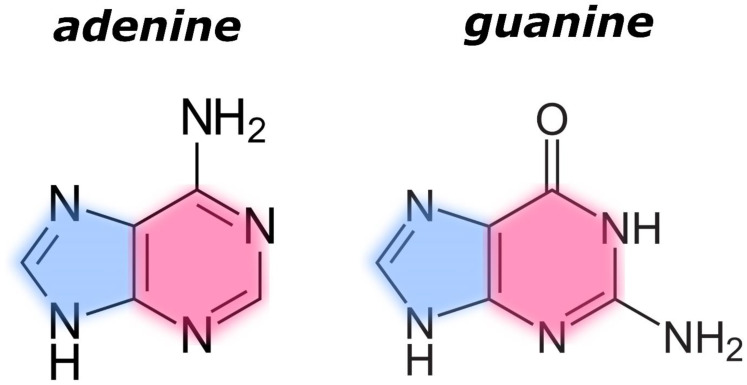
Molecular structures of adenine and guanine. In each case, the pyrimidine and imidazole ring components of the molecule have been indicated using red and blue highlighting, respectively.

Purines are nitrogen-bearing heterocyclic molecules whose backbone is composed of a pyrimidine ring fused to an imidazole ring. The purine derivatives adenine and guanine (Figure 1) are components of the nucleic acids DNA and RNA, which are central molecules in the evolutionary, hereditary, and genetic processes of all living organisms on Earth. They are also participants in cellular metabolic processes, being integral components of energy-carrying molecules such as adenosine triphosphate (ATP), guanosine triphosphate (GTP), nicotinamide adenine dinucleotide (NAD), and flavine adenine dinucleotide (FAD). Therefore, from the perspective of astrobiology, adenine and guanine are complex organic molecules that are essential for the emergence of life [1] and so their detection in interstellar regions represents one of the most sought-after results in contemporary astrobiological and astrochemical research.

Purines are ubiquitous in terrestrial environments [2] but have yet to be detected in observations of the interstellar medium [3]. Despite this, there is a large body of evidence that suggests that the formation of several nucleobases (including purines) is feasible under conditions relevant to the dense interstellar medium [4,5,6]. Most recently, Oba et al. [4] have demonstrated that a number of purine-based nucleobases, including adenine, xanthine, and hypoxanthine, can be synthesised through the ultraviolet (Lyman-α) irradiation of interstellar ice analogues containing the most abundant molecules found in dense molecular clouds, namely H_2_O, NH_3_, CO, and CH_3_OH. Further evidence for the extraterrestrial origins of purines has resulted from the analysis of meteorites, in which a number of nucleobases have been detected [7,8,9,10]. For example, adenine and guanine have been directly identified in fragments of the Murchison, Murray, and Tagish Lake meteorites [10]. Interestingly, purines that are exceedingly rare on Earth (such as 2,6-diaminopurine and 6,8-diaminopurine) have also been detected in meteorites [8], thus strengthening their postulated extraterrestrial origin.

Saladino et al. [11] also performed experiments to test the plausibility of nucleobase formation in meteorites, in which they heated samples of the Murchison meteorite to a temperature of 140 °C in the presence of formamide after which the formation of purine and adenine, together with a number of other nucleobases, was noted. The terrestrial synthesis of purines starting from HCN has also been investigated by previous studies [12], but formamide represents one of the most well-studied molecules in the abiogenic synthesis of nucleobases due to the fact that its polymerisation is known to yield adenine, guanine, thymine, uracil, and cytosine under conditions relevant to prebiotic chemistry [13,14,15,16,17,18]. Furthermore, it is also known to be present in the interstellar medium, having been detected in the protostellar disk of the young stellar object W33A [19].

The evidence for the efficient synthesis of nucleobases (including purines) under extraterrestrial environmental conditions thus contrasts with the hitherto lack of any direct observation of these species in the interstellar medium. Since interstellar icy grain mantles in the cores of dense molecular clouds are considered to be the ‘molecular factories’ of the cosmos [1], the identification of biomolecules such as purines in these environments is a much sought-after result. Such an identification must rely on infrared absorption spectroscopy, as the rotational motion needed for the more traditionally used radio astronomy is restricted in the solid phase [20]. However, the detection of purines (as well as other complex organic molecules) via infrared absorption spectroscopy presents several challenges, many of which were discussed in the works of Rosa et al. [21,22]. One of the more noteworthy challenges is the fact that the infrared absorption spectrum of an astronomical target is densely populated by absorption bands: many of these bands are attributable to simple, volatile molecules that condense on interstellar dust grains in dense clouds to form an icy mantle, such as H_2_O, NH_3_, CH_4_, CO, and CH_3_OH [3,23,24], although some of these bands are also likely attributable to complex organic molecules that are synthesised as a result of the processing of these simple molecular ices by galactic cosmic rays and Lyman-α photons [1,24,25,26,27,28,29,30].

As such, it is likely that the bands due to ubiquitous simple, volatile molecules may obscure those of lower concentration complex organic molecules (such as purine nucleobases) in several key regions of the infrared absorption spectra of astronomical targets. However, it is possible that the most promising region of the infrared spectrum for the detection of interstellar purines has yet to be identified. This work therefore describes a study of the infrared spectra of purine nucleobases linked to terrestrial biochemical processes under conditions analogous to those found in the interstellar medium with the aim of identifying any characteristic absorption features that may exist in regions of the infrared absorption spectra of astronomical targets that are less densely populated by absorption bands. In particular, the infrared absorption spectra of adenine, guanine, and a mixture of these two nucleobases, both in neat form and embedded within an interstellar ice analogue composed of H_2_O:NH_3_:CH_4_:CO:CH_3_OH (10:1:1:1:1), were studied. It is anticipated that the data presented herein will serve as a reference for observations conducted using high-sensitivity and high-resolution instruments such as the recently launched *James Webb Space Telescope* [31].

## 2. Materials and Methods

### 2.1. Experimental Apparatus

Experiments were performed using the Ice Chamber for Astrophysics-Astrochemistry (ICA) at the HUN-REN Institute for Nuclear Research (Atomki) in Debrecen, Hungary. The ICA is an experimental set-up dedicated to the study of the infrared spectroscopy and radiation chemistry of interstellar ice analogues [32,33]. The set-up consists of an ultrahigh-vacuum stainless-steel chamber having an operational base pressure of 10^−9^ mbar which is maintained by the combined action of a scroll pump and a turbomolecular pump. Within the centre of the chamber is a gold-coated oxygen-free high-conductivity copper sample holder into which a maximum of four infrared-transparent ZnSe sample substrates may be mounted. The cold finger of a closed-cycle helium cryostat is held in contact with the sample holder and allows it and the substrates to be cooled to 20 K; although an operational temperature range of 20–300 K is available.

The ICA is equipped with a mid-infrared spectrophotometer (Thermo Nicolet Nexus 670) having a spectral range of 4000–650 cm^−1^ and a nominal resolution of 1 cm^−1^. Infrared spectroscopic studies are performed in transmission absorption mode with the infrared beam maintained orthogonal to the plane of the surface of the sample substrates and being detected by an external mercury–cadmium–telluride (MCT) detector. A quadrupole mass spectrometer (Pfeiffer QME200) is also attached to the ICA and allows for the in situ measurement of the gas-phase composition of the chamber.

### 2.2. Preparation and Quantification of Purines Embedded in Interstellar Ice Analogues

Powdered adenine (≥99% purity) and guanine (≥98% purity) were purchased from Sigma-Aldrich and were used to prepare diluted solutions. Adenine was diluted in a solution of 60.8% *v*/*v* ethanol in water, while guanine and the mixture of adenine and guanine were diluted in a 0.1 M solution of NaOH. In each case, a diluted solution with a final concentration of 1.5 mg mL^−1^ was prepared from which approximately 400 μL was dropped onto a ZnSe substrate using a clean pipette. The ZnSe substrates were then heated to 100 °C until total evaporation of the solvent, thereby producing a ‘grainy’ solid film of crystalline purine nucleobases deposited on the surface of the substrates.

The three ZnSe substrates with purine nucleobases (i.e., neat adenine, neat guanine, and the mixture of the two) deposited on their surfaces were then mounted into the sample holder of the ICA apparatus and the chamber was evacuated to base pressure overnight. Once at base pressure, mid-infrared absorption spectra of the purines were acquired at 300 K using a control ZnSe sample substrate which only had solvent dropped on it before being heated to 100 °C as a background. The sample holder and substrates were subsequently cooled to 20 K and another set of mid-infrared absorption spectra was acquired. At 20 K, interstellar ice analogues were deposited onto the cooled substrates via the background deposition of relevant gases or vapours. According to the current inventory of interstellar ice components, the major volatiles with infrared bands in the same region as those expected for the purine nucleobases (~5.5–14 μm) are H_2_O (6.0 and ~13.6 μm), NH_3_ (9.0 μm), CH_4_ (7.67 μm), CO (4.67 μm), and CH_3_OH (6.85 and 9.75 μm) [31,34,35]. Accordingly, these volatiles were selected as the components of our interstellar ice analogues. First, H_2_O vapour from a de-ionised H_2_O sample that had been de-gassed via multiple iterations of the freeze–pump–thaw cycle was introduced into the chamber to deposit a layer of H_2_O ice. This was followed by the co-deposition of a 1:1 mixture of NH_3_ and CH_4_, which in turn was followed by the co-deposition of a 1:1 mixture of CO and CH_3_OH.

The choice to deposit the ice layer-by-layer was motivated by the actual structure of interstellar ices, which are known to be organised into a lower polar layer rich in molecules formed as a result of the surface-catalysed hydrogenation of heteroatoms (e.g., H_2_O, NH_3_, CH_4_) and an upper apolar layer formed by the catastrophic condensation of CO and its subsequent solid-phase hydrogenation to CH_3_OH [24,36]. The separate deposition of H_2_O and NH_3_ also has the advantage of preventing any gas-phase reactions between these species, which have been reported to yield hydrates of NH_3_ and NH_4_OH [37]. The final result of the deposition procedure was a series of layered H_2_O:NH_3_:CH_4_:CO:CH_3_OH (10:1:1:1:1) ices having a thickness of between 1.8–3.5 μm deposited on top of interstellar purine analogues whose infrared absorption spectra were subsequently acquired. It is to be noted that the spectrum of the layered ice deposited on top of the nucleobases is virtually identical to the sum of the spectra of the individual nucleobases and of the mixture of volatiles. This therefore suggests that our experiments should produce infrared data which are representative of infrared signals produced by actual interstellar icy grain mantles. The composition of the interstellar ice analogues was confirmed by measuring the column densities *N* (molecules cm^−2^) of each component using the equation:

(1) $$N=ln(10)\frac{\int_{\nu_{1}}^{\nu_{2}}Abs\;d\nu }{A}$$

where the integral in the numerator corresponds to the area of an infrared absorption band characteristic to a particular molecular species and *A* is the integrated band strength constant associated with that band. Values for the integrated band strengths of the absorption bands measured to assign the different ice components and quantify their abundance were taken from the work of Bouilloud et al. [38].

The molecular column densities of the purine molecules were quantified by measuring the area of the so-called ‘α-band’, which is a series of absorption features characteristic to nucleobases between 3600–1970 cm^−1^ in the case of adenine and 3700–2120 cm^−1^ in the case of guanine, for which the integrated band strength constants are known [39,40]. The final column density ratios of the H_2_O:NH_3_:CH_4_:CO:CH_3_OH:purine interstellar ice analogues were 10:1:1:1:1:0.2 in the case of adenine and 10:1:1:1:1:0.4 in the case of guanine. It was not possible to calculate the column density ratios of adenine and guanine in the mixed purine ice analogue due to the overlap of the respective α-bands.

## 3. Results

### 3.1. Mid-Infrared Absorption Spectra of Purines at 300 and 20 K

**Figure 2:**
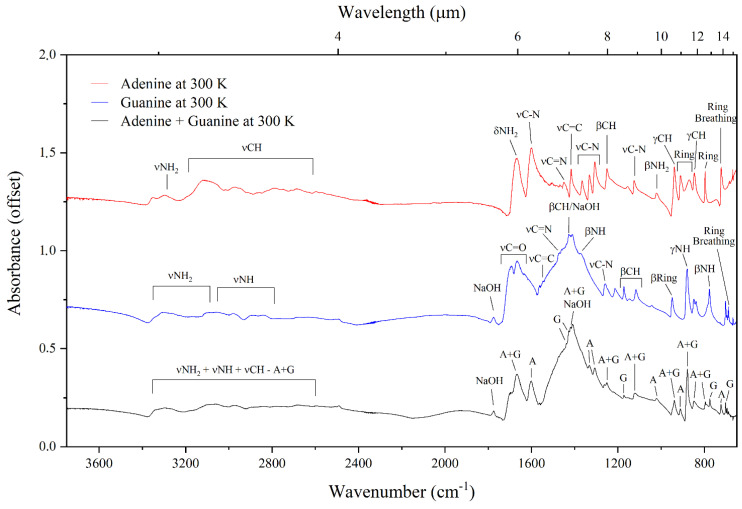
Mid-infrared absorption spectra of neat adenine, neat guanine, and the adenine–guanine mixture at 300 K.

**Figure 3:**
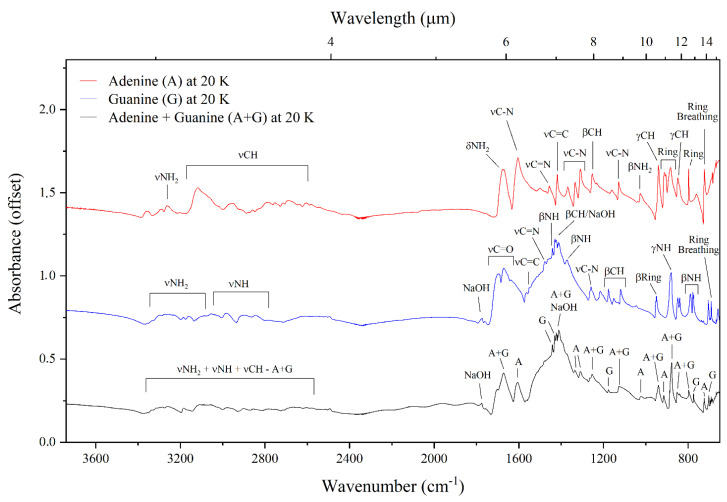
Mid-infrared absorption spectra of neat adenine, neat guanine, and the adenine–guanine mixture at 20 K.

The mid-infrared absorption spectra of adenine, guanine, and the adenine–guanine mixture at 300 and 20 K are shown in Figure 2 and Figure 3, respectively. The assignments of the bands observed in these absorption spectra, which are based on assignments made by previous studies [39,40,41,42,43,44,45,46,47,48], are listed in Table 1. It is to be noted that our experimental methodology resulted in the preparation of crystalline nucleobase samples; however, any nucleobases synthesised under the cold conditions of dense interstellar clouds would be expected to have an amorphous structure. In spite of this difference, previous studies have demonstrated that the infrared absorption spectra of amorphous and crystalline phases are very similar, with the majority of bands being in the same position. For instance, Chen et al. [49] showed that the spectrum of guanine is identical in both phases, with the exception of the band at about 3500 cm^−1^ which is broader in the amorphous phase.

With regards to temperature-dependent differences, a few dissimilarities in the appearances of the spectra at 20 and 300 K are noticeable, which therefore suggest that the infrared absorption spectra of adenine and guanine in quiescent molecular clouds (where temperatures are as low as 10–20 K) will differ to those of similar molecules after the collapse of the molecular cloud, where temperatures may easily reach 300 K. For instance, by comparing the spectra of adenine collected at 300 and 20 K, it is possible to note that the absorption bands between 3500–2500 cm^−1^ are visibly sharper at the latter temperature. These bands correspond to the νNH_2_ and the νCH modes of adenine. The bands assigned to the β-ring (912 cm^−1^) and the ring deformation (883 cm^−1^) modes are also sharper in the 20 K spectrum compared to the 300 K spectrum. Similar to the case of adenine, the bands in the 3500–2500 cm^−1^ region of the infrared absorption spectrum of guanine are sharper and better defined at 20 K compared to the 300 K spectrum. These bands correspond to the νNH_2_ and νNH modes of guanine. Moreover, the band assigned to the βNH mode (1441 cm^−1^) is only apparent in the 20 K spectrum. Furthermore, the absorption spectrum of guanine at 300 K exhibits a single band at 776 cm^−1^, while in the 20 K spectrum two bands are observed in this region at 789 and 778 cm^−1^. We note that, although the βCH mode of guanine is expected at 1430 cm^−1^ and a band is indeed observed in this region of the spectra of the guanine sample and the adenine–guanine mixture at both 300 and 20 K, we attribute this band to NaOH, which was used as a solvent in the preparation of both these samples. An additional absorption band attributable to NaOH was also observed at 1777 cm^−1^.

**Figure 4:**
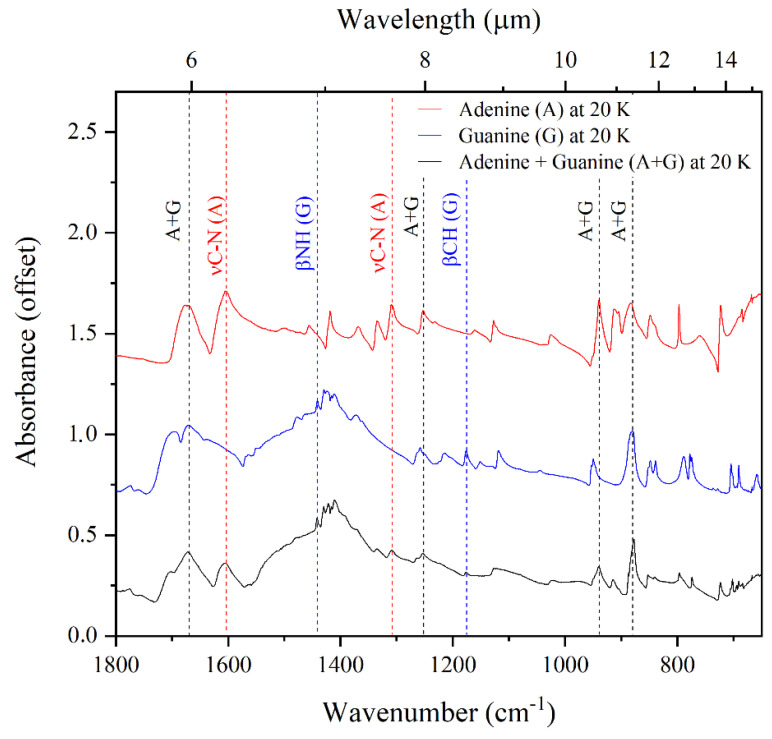
The fingerprint region (1800–650 cm^−1^) of the mid-infrared absorption spectra of neat adenine, neat guanine, and the adenine–guanine mixture at 20 K. The red lines compare the positions of bands solely attributable to adenine in the spectra of neat adenine and the adenine–guanine mixture. The blue lines compare the positions of bands solely attributable to guanine in the spectra of neat guanine and the adenine–guanine mixture. The black lines compare the positions of bands in the spectra of the pure nucleobases to that of the mixture.

In the case of the adenine–guanine mixture, the only noticeable difference between the 300 and 20 K absorption spectra is the increased sharpness of the bands in the 3500–2500 cm^−1^ region of the latter. Interestingly, in both spectra, it is possible to identify bands that can be attributed solely to either adenine or guanine (respectively, labelled as (A) and (G) in Figure 2 and Figure 3), as well as bands that arise due to the blending of coincident absorption features of these molecules (labelled as (A + G) in Figure 2 and Figure 3). Our results demonstrate that there is no measurable shift in the positions of the infrared absorption bands in the spectra of the neat purines compared to that of the adenine–guanine mixture (Figure 4); indeed, these spectra are virtually identical.

### 3.2. Mid-Infrared Absorption Spectra of Purines Embedded within Interstellar Ice Analogues at 20 K

**Figure 5:**
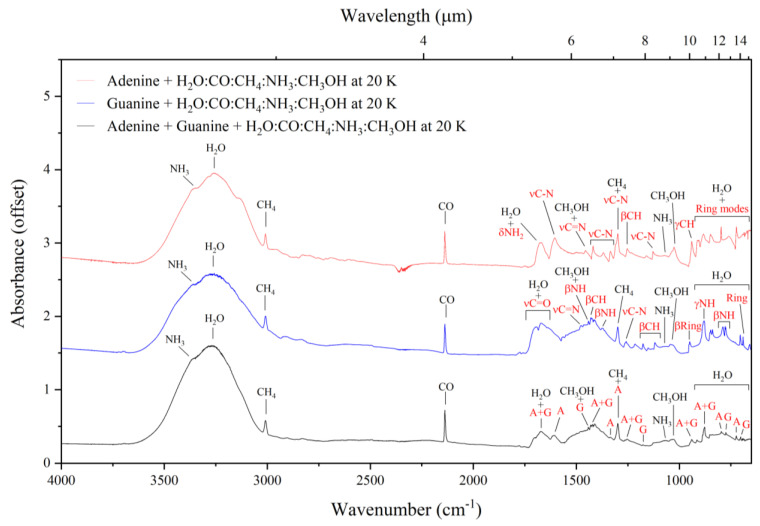
Mid-infrared absorption spectra of adenine, guanine, and the adenine–guanine mixture embedded within interstellar ice analogues at 20 K. Absorption features attributable to the purines are indicated in red.

The mid-infrared spectra of interstellar ice analogues composed of H_2_O:NH_3_:CH_4_:CO:CH_3_OH (10:1:1:1:1) deposited on top of adenine, guanine, and the adenine–guanine mixture at 20 K are shown in Figure 5, and a full assignment of each of the bands observed in these spectra is given in Table 2. Our analyses of these spectroscopic data complement and extend the previous results of Rosa et al. [21,22], who suggested that three pairs of almost coincident bands in the spectra of adenine and guanine could be used as generic mid-infrared spectral signatures of purine nucleobases. These pairs of bands are: (i) 1371 (adenine: νCN and βCH) and 1373 cm^−1^ (guanine: βNH, βCH, and νCN), (ii) 1257 (adenine: βCH and βNH) and 1264 cm^−1^ (guanine: νCN), and (iii) 940 (adenine: γCH) and 950 cm^−1^ (guanine: β-ring and γC=O).

However, the results of this present study demonstrate that when adenine, guanine, or an adenine–guanine mixture are embedded within an interstellar ice analogue, many of the infrared absorption bands attributable to the purine nucleobases are obscured by other absorption features attributable to the volatile components of the ice (Figure 5). Nonetheless, it is still possible to distinguish a number of these absorption bands, which are highlighted in red in Figure 5. Considering the spectrum of the adenine–guanine mixture embedded within an interstellar ice analogue, five absorption bands attributable to adenine (at 1606, 1336, 1299, 797, and 724 cm^−1^), four bands attributable to guanine (at 1442, 1178, 774, and 702 cm^−1^), and five bands resulting from the blending of the adenine and guanine absorption features (at 1670, 1422, 1255, 940, and 878 cm^−1^) are evident. As the aim of this study is the identification of interstellar spectral signatures attributable to purine nucleobases in a general sense, it is these latter five absorption bands that will be the focus of the remainder of this discussion.

**Figure 6:**
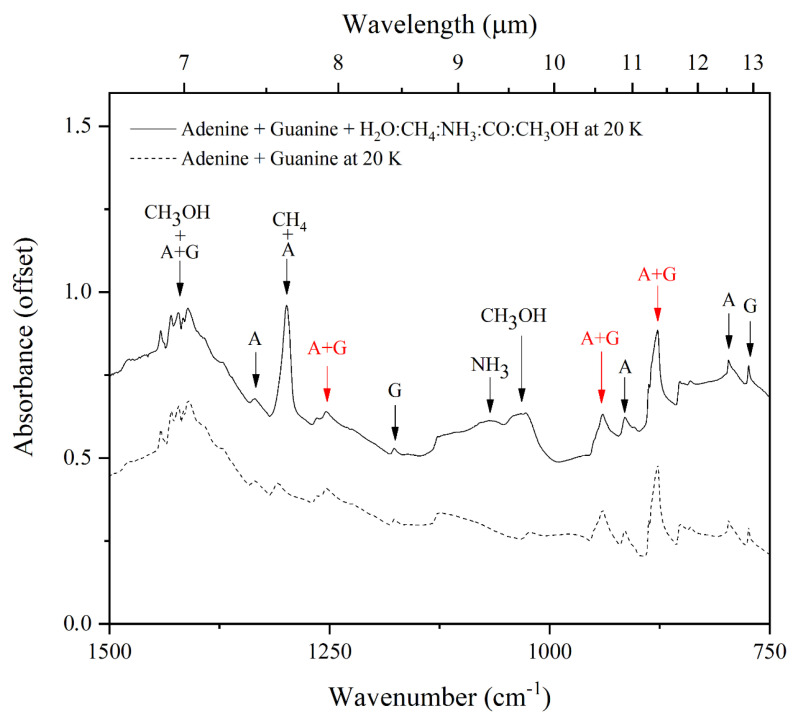
The bands highlighted by red arrows indicate the three bands that are proposed as mid-infrared spectral signatures of adenine and guanine in interstellar ices.

It should be noted that, although the absorption feature at 1670 cm^−1^ (assigned to the νC=O and the βNH_2_ modes of guanine and the δ_scis_NH_2_ mode of adenine; Table 2) is distinguishable in our laboratory-generated spectrum, it is likely that it will not be evident in mid-infrared observations of dense interstellar clouds due to it being obscured by the intense ν_2_ mode of H_2_O ice [50,51]. A similar argument may be invoked for the absorption feature at 1422 cm^−1^ (assigned to the βCH mode of guanine and the νC=C, νCN, and βCH modes of adenine), which is partially concealed by the CH bending mode of CH_3_OH [52,53]. For these reasons, we discount these two absorption bands as possible mid-infrared spectral signatures of nucleobases in dense interstellar regions which are rich in H_2_O and CH_3_OH ice. Conversely, the other three purine nucleobase bands at 1255, 940, and 878 cm^−1^ do not coincide with absorption features attributable to the most common molecules in the icy cosmos (Figure 6) and are thus promising candidates for the detection of interstellar purines.

## 4. Astrochemical Implications

From the perspective of astrobiology and prebiotic chemistry, the nucleobases adenine and guanine are likely to be co-synthesised in extraterrestrial environments due to their related abiotic synthesis pathways and their common precursor molecules [6,54,55,56,57,58,59,60,61,62]. Indeed, both these species have been detected in fragments of the Murchison, Murray, and Tagish Lake meteorites [10]. As such, spectroscopic surveys of interstellar targets aiming to detect these purine-based nucleobases should be supported by laboratory-generated data of adenine–guanine mixtures acquired under conditions relevant to the interstellar medium. In this present study, we have acquired the mid-infrared absorption spectra of neat adenine, neat guanine, and an adenine–guanine mixture at 300 and 20 K and, for the first time, of these nucleobases embedded within ices analogous to those expected in dense interstellar clouds at 20 K. 

The interstellar ice analogues used in this study were composed of H_2_O, NH_3_, CH_4_, CO, and CH_3_OH, which represent the most abundant icy species in the cosmos [3,23,24] and are thus the most likely candidates whose absorption bands could potentially obscure those of interstellar purine nucleobases. However, our results have demonstrated that three mid-infrared absorption bands show great promise as key spectroscopic signatures to detect mixtures of adenine and guanine in the interstellar medium due to the fact that they are evident in acquired spectra even when comparatively large abundances of interstellar ice components are also present (Table 3). Two of these bands (those at 1257 and 940 cm^−1^ for adenine and 1264 and 950 cm^−1^ for guanine) were identified and discussed in our previous work [21,22]. However, in this present study, we have been able to identify a third band at 878 cm^−1^ in the spectrum of the adenine–guanine mixture which was assigned to a blending of bands from the individual molecules. Furthermore, it is to be noted that these three bands are distinguishable from other absorption features attributable to nitrogen-bearing polycyclic aromatic hydrocarbons (N-PAHs), which are also believed to be relatively abundant in the interstellar medium [28,63,64]. This is because the purine infrared absorption band at around 945 cm^−1^ in particular, occupies a region of the spectrum wherein there is minimal overlap with bands attributable to N-PAHs [65].

Infrared features of interstellar ices have been reported in the literature ever since space-borne telescopes, such as the *Infrared Space Observatory* and the *Spitzer Space Telescope*, made their first observations. The data being presently generated by the recently launched *James Webb Space Telescope* has allowed for the detection of hitherto unknown infrared bands attributable to various molecular components of interstellar ices, thereby increasing the inventory of known ice components [31,34,35]. However, none of these molecular components presents infrared spectroscopic features at similar wavenumbers to those proposed as signatures of interstellar purines in the region around 1255, 940, and 878 cm^−1^ (7.96, 10.63, and 11.38 μm). However, laboratory-generated spectroscopic data of complex organic molecules has demonstrated that some species do indeed present bands at similar wavenumbers as the 878 cm^−1^ (11.38 μm) purine band, among them being the CH_3_ rocking mode of amorphous acetone (871 cm^−1^, 11.48 μm) and the CCO stretching mode of amorphous ethanol (879 cm^−1^, 11.37 μm) [66,67]. As such, if the three bands proposed in this paper as a spectroscopic signature for interstellar purines are indeed observed in an interstellar ice, then the band at 878 cm^−1^ (11.38 μm) may be potentially attributed to either the purines, acetone, or ethanol and it would be practically impossible to quantify the contribution of each individual species to the total signal of this particular band.

We are, however, nonetheless able to conclude that the simultaneous observation of weak mid-infrared absorption bands at approximately 1255, 940, and 878 cm^−1^ (7.96, 10.63, and 11.38 μm) in spectroscopic surveys of astronomical targets may be a promising indicator of the presence of purines. In order to identify optically weak bands, instruments with moderate-to-high spectral resolutions and high signal-to-noise (S/N) ratios are required. For reasons more fully discussed in the works of Rosa et al. [21,22], it was not possible to observe optically weak bands using the instruments onboard the *Infrared Space Observatory* or the *Spitzer Space Telescope* which, until the launch of the *James Webb Space Telescope*, were the most recent space-borne observatories that collected observational data in the infrared spectral range. Conversely, the Mid-Infrared Instrument (MIRI) onboard the *James Webb Space Telescope* is able to collect observational data with very high resolution and within shorter exposure times. To compare, the MIRI has a resolving power ranging from 1790–3750, which is thirty times greater than that of the Infrared Spectrograph (IRS) onboard the *Spitzer Space Telescope*. Therefore, the *James Webb Space Telescope* has the required resolving power to observe optically weak bands such as those proposed as potential signatures of interstellar purines in this work. 

It is important to note at this point that all three bands proposed herein as mid-infrared spectroscopic signatures of interstellar purines have calculated integrated molar absorptivity values greater than 4 km mol^−1^ (Table 3), as was recently reported by Iglesias-Groth and Cataldo [68]. The integrated molar absorptivity, *ψ*, is a spectrochemical parameter that indicates how strongly a molecular species absorbs, and therefore attenuates, light across a defined wavenumber range. It is equivalent to the integral of the more commonly used molar extinction coefficient, *ε*, across a defined wavenumber range and is related to the integrated area of an absorption band as measured from an acquired mid-infrared spectrum as follows:

(2) $$\psi =\frac{1}{lc}\int_{\nu_{1}}^{\nu_{2}}Abs\;d\nu$$

where *l* refers to the pathlength of the infrared light and *c* is the concentration of the absorbing molecule. Given that all three of the bands in question satisfy the condition *ψ* > 4 km mol^−1^ [68], then they should all be readily detectable in observational spectra acquired by the *James Webb Space Telescope*. 

**Table 3:** Mid-infrared absorption bands proposed to be promising spectroscopic signatures for the presence of adenine and/or guanine in interstellar ices. Reported band peak positions have an uncertainty of ±2 cm^−1^. Values for the integrated molar absorptivities (*ψ*) are taken from the work of Iglesias-Groth and Cataldo [68].

*ν* (cm^−1^)	*λ* (μm)	Assignment ^†^	*ψ* (km mol^−1^)
1255	7.97	βCH (A), βNH (A), and νCN (G)	23 (A); 16 (G)
940	10.64	γCH (A), β-ring (G), and γC=O (G)	21 (A); 10 (G)
878	11.39	ring deformation (A) and γNH (G)	4.1 (A); 19 (G)

^†^ Band assignments to adenine and guanine are indicated by (A) and (G), respectively.

## 5. Conclusions

Adenine and guanine play central roles in the biochemistry that support life on Earth, most prominently in genetic and metabolic processes. Despite the existence of a number of favourable synthesis routes towards the formation of purine nucleobases (including adenine and guanine) in extraterrestrial environments, as well as their identification in several meteorites, such molecules have yet to be directly detected in spectroscopic observations of interstellar space. In this present study, we have analysed the mid-infrared absorption spectra of adenine, guanine, and a mixture of the two embedded within an interstellar ice analogue composed of the most abundant volatile molecules in molecular clouds (i.e., H_2_O, NH_3_, CH_4_, CO, and CH_3_OH). 

Our results have demonstrated that three absorption bands in the spectrum of the adenine–guanine mixture embedded within an interstellar ice analogue may prove useful as mid-infrared spectroscopic signatures of purine derivatives in interstellar ices, as they do not coincide or overlap with any of the intense absorption features attributable to the volatile components of the interstellar ice analogue. These bands are located at 1255, 940, and 878 cm^−1^ (band peak positions in our laboratory spectra have an uncertainty of ±2 cm^−1^) and have integrated molar absorptivities that are sufficiently high as to make their detection by high-sensitivity and high-resolution instruments (such as the recently launched *James Webb Space Telescope*) possible. The consideration of bands common to different purine nucleobases is advantageous since the similarity of the abiotic synthesis pathways towards these structurally related molecules means that many purines are likely co-synthesised in interstellar environments, and thus searching for these common absorption bands will increase the likelihood of a positive interstellar detection. 

Indeed, the detection of nucleobases in extraterrestrial environments is an important step forward in understanding the formation and cosmic distribution of these molecules prior to their incorporation in biological and biochemical systems. Laboratory studies, such as the one presented here, provide new data that can help in the detection of these complex organic molecules in interstellar space, thereby providing novel insights into the discussion on the endogenous vs. exogenous syntheses of the first biomolecules and the origins of life on Earth and possibly elsewhere.

## Figures and Tables

**Table 1:** Mid-infrared band assignments of interstellar analogues of neat adenine, neat guanine, and the adenine–guanine mixture at 20 K.

Adenine			Guanine			Adenine–Guanine Mixture ^†^		
ν (cm^−1^)	Assignment	Reference	ν (cm^−1^)	Assignment	Reference	ν (cm^−1^)	Assignment	Reference
3354	νNH	[45]	3159	ν_sym_NH_2_	[46]	3284	νNH_2_ (A)	[45]
3286	νNH_2_	[45]	2896	νNH	[46]	3257	νNH_2_ (A)	[47]
3263	νNH_2_	[47]	2841	νNH	[41,42,46]	3184	νNH_2_ (G)	[46]
3117	νCH	[45,47]	1694	νC=O and βNH_2_	[41,46]	2978	νCH (G)	[45,47]
2950	νCH	[45,47]	1670	νC=O and βNH_2_	[40,41,46]	2901	νNH (G)	[46]
2788	νCH	[39,47]	1551	νC=C	[41,46]	2854	νNH (G)	[41,42,46]
2686	νCH	[39,47]	1480	νCN and νC=N	[41]	2782	νCH (A)	[39,47]
1675	δ_scis_NH_2_	[39,45,47]	1441	βNH	[40,41,42]	1672	νC=O (G), βNH_2_ (G), and δ_scis_NH_2_ (A)	[39,40,41,45,46,47]
1604	νCN and νCC	[39,43,44,45,47,48]	1429	βCH and NaOH	[41]	1605	νCN (A) and νCC (A)	[39,43,44,45,47,48]
1499	ν-ring	[45]	1372	βNH, βCH, and νCN	[40,41,42]	1442	βNH (G)	[40,41,42]
1456	νC=N and βCH	[43,44,47,48]	1258	νCN	[40,41]	1430	βCH (G) and NaOH	[41]
1419	νC=C, νCN, and βCH	[39,43,44,45,47,48]	1214	νCNH_2_	[41]	1422	νC=C (A), νCN (A), and βCH (A)	[39,43,44,45,47,48]
1369	νCN and βCH	[39,43,44,45,48]	1176	βCH	[41,42]	1336	νCN (A) and βCH (A)	[39,43,44,45,47,48]
1334	νCN and βCH	[39,43,44,45,47,48]	1151	βCH	[41]	1309	νCN (A)	[39,43,44,45,48]
1309	νCN	[39,43,44,45,48]	1119	βCH	[40,42]	1253	βCH (A), βNH (A), and νCN (G)	[39,40,41,43,44,45,47]
1253	βCH and βNH	[39,43,44,45,47,48]	950	β-ring and γC=O	[42]	1178	βCH (G)	[41,42]
1128	νCN	[39,43,44,45,47,48]	880	γNH	[41]	1127	νCN (A) and βCH (G)	[39,40,43,44,45,47,48]
1025	β_rock_NH_2_ and νC=N	[39,43,44,45,47,48]	848	νCC and β-ring	[41,42]	1023	β_rock_NH_2_ (A) and νC=N (A)	[39,43,44,45,47,48]
940	γCH	[43,44,45,47,48]	789	βNH	[41]	940	γCH (A), β-ring (G), and γC=O (G)	[42,43,44,45,48]
913	Β-ring and νC=C	[43,44,45,47,48]	778	βNH	[41,42]	915	β-ring (A) and νC=C (A)	[43,44,45,47,48]
883	ring deformation	[45]	705	β-ring	[41]	877	ring deformation (A) and γNH (G)	[41,45]
848	γCH	[45]	691	ring breathing	[41]	852	γCH (A), νCC (G), β-ring (G)	[41,42,45]
797	ring torsion	[45]				797	ring torsion (A) and βNH (G)	[41,45]
723	ring breathing	[43,45,47,48]				774	βNH (G)	[41,42]
						724	ring breathing (A)	[43,45,47,48]
						702	β-ring (G)	[41]
						691	ring breathing (G)	[41]

^†^ Band assignments to adenine and guanine are indicated by (A) and (G), respectively.

**Table 2:** Mid-infrared band assignments of adenine, guanine, and the adenine–guanine mixture embedded within interstellar ice analogues at 20 K. Band assignments due to individual molecules are indicated in brackets, where (A) and (G) respectively refer to adenine and guanine.

Adenine + H_2_O:NH_3_:CH_4_:CO:CH_3_OH			Guanine + H_2_O:NH_3_:CH_4_:CO:CH_3_OH		Adenine–Guanine Mixture + H_2_O:NH_3_:CH_4_:CO:CH_3_OH		
ν (cm^−1^)	Assignment		ν (cm^−1^)	Assignment	ν (cm^−1^)	Assignment	
3362	ν_3_ (NH_3_)		3362	ν_3_ (NH_3_)	3362	ν_3_ (NH_3_)	
3257	ν_3_ (H_2_O)		3257	ν_3_ (H_2_O)	3270	ν_3_ (H_2_O)	
3009	ν_3_ (CH_4_)		3009	ν_3_ (CH_4_)	3009	ν_3_ (CH_4_)	
2138	ν (CO)		2913	combination modes (CH_3_OH)	2913	combination modes (CH_3_OH)	
1671	δ_scis_NH_2_ (A) and ν_2_ (H_2_O)		2830	ν_3_ (CH_3_OH)	2830	ν_3_ (CH_3_OH)	
1605	νCN (A) and νCC (A)		2138	ν (CO)	2138	ν (CO)	
1456	νC=N (A), βCH (A), and ν_10_ (CH_3_OH)		1692	νC=O (G), βNH_2_ (G), and ν_2_ (H_2_O)	1670	νC=O (G), βNH_2_ (G), δ_scis_NH_2_ (A), and ν_2_ (H_2_O)	
1419	νC=C (A), νCN (A), and βCH (A)		1670	νC=O (G) and ν_2_ (H_2_O)	1606	νCN (A), νCC (A), and ν_2_ (H_2_O)	
1369	νCN (A) and βCH (A)		1478	νC=N (G) and νCN (G)	1442	βNH (G) and ν_10_ (CH_3_OH)	
1334	νCN (A) and βCH (A)		1441	βNH (G) and ν_10_ (CH_3_OH)	1422	βCH (G), νC=C (A), νCN (A), and βCH (A)	
1299	νCN (A) and ν_4_ (CH_4_)		1430	βCH (G)	1336	νCN (A) and βCH (A)	
1253	βCH (A) and βNH (A)		1375	βNH (G), βCH (G), and νCN (G)	1299	νCN (A) and ν_4_ (CH_4_)	
1128	νCN (A)		1299	ν_4_ (CH_4_)	1255	βCH (A), βNH (A), and νCN (G)	
1069	ν_2_ (NH_3_)		1258	νCN (G)	1178	βCH (G)	
1026	ν_8_ (CH_3_OH)		1214	νCNH_2_ (G)	1070	ν_2_ (NH_3_)	
940	γCH (A)		1176	βCH (G)	1035	ν_8_ (CH_3_OH)	
910	β-ring (A) and νC=C (A)		1153	βCH (G)	940	γCH (A), β-ring (G), and γC=O (G)	
883	ring deformation (A)		1118	βCH (G)	915	β-ring (A) and νC=C (A)	
848	γCH (A)		1070	ν_2_ (NH_3_)	878	ring deformation (A) and ν_L_ (H_2_O)	
797	ring torsion (A) and ν_L_ (H_2_O)		1038	ν_8_ (CH_3_OH)	797	ring torsion (A) and ν_L_ (H_2_O)	
723	ring breathing (A) and ν_L_ (H_2_O)		950	β-ring (G) and γC=O (G)	774	βNH (G) and ν_L_ (H_2_O)	
			880	γNH (G)	724	ring breathing (A) and ν_L_ (H_2_O)	
			848	νCC (G) and β-ring (G)	702	β-ring (G) and ν_L_ (H_2_O)	
			799	βNH (G) and ν_L_ (H_2_O)	691	ring breathing (G) and ν_L_ (H_2_O)	
			778	βNH (G) and ν_L_ (H_2_O)			
			705	β-ring (G) and ν_L_ (H_2_O)			
			691	ring breathing (G) and ν_L_ (H_2_O)			

## Data Availability

All data discussed herein will be made available to any interested party upon reasonable request of one of the corresponding authors.
